# BloodPAC Data Commons for Liquid Biopsy Data

**DOI:** 10.1200/CCI.20.00179

**Published:** 2021-04-30

**Authors:** Robert L. Grossman, Jonathan R. Dry, Sean E. Hanlon, Donald J. Johann, Anand Kolatkar, Jerry S. H. Lee, Christopher Meyer, Lea Salvatore, Walt Wells, Lauren Leiman

**Affiliations:** ^1^ Center for Translational Data Science, University of Chicago, Chicago, IL; ^2^ Open Commons Consortium, Chicago, IL; ^3^ Tempus Labs, Chicago, IL; ^4^ National Cancer Institute, Bethesda, MD; ^5^ University of Arkansas for Medical Sciences, Little Rock, AR; ^6^ Convergent Science Institute in Cancer, Michelson Center for Convergent Bioscience, University of Southern California, Los Angeles, CA; ^7^ Lawrence J. Ellison Institute for Transformative Medicine, University of Southern California, Los Angeles, CA; ^8^ Progressive Insurance, Cleveland, OH; ^9^ BloodPAC Consortium, Chicago, IL

## Abstract

The Blood Profiling Atlas in Cancer (BloodPAC) Data Commons (BPDC) is being developed and is operated by the public-private BloodPAC Consortium to support the liquid biopsy community. It is an interoperable data commons with the ultimate aim of serving as a recognized source of valid scientific evidence for liquid biopsy assays for industry, academia, and standards and regulatory stakeholders.

**METHODS:**

The BPDC is implemented using the open source Gen3 data commons platform (https://gen3.org). In particular, the BPDC Data Exploration Portal, BPDC Data Submission Portal, the BPDC Workspace Hub, and the BloodPAC application programming interface (API) were all automatically generated from the BloodPAC Data Model using the Gen3 data commons platform. BPDC uses Gen3's implementation of the data commons framework services so that it can interoperate through secure, compliant APIs with other data commons using data commons framework service, such as National Cancer Institute's Cancer Research Data Commons.

**RESULTS:**

The BPDC contains 57 studies and projects spanning more than 4,100 cases. This amounts to 5,700 aliquots (blood plasma, serum, or a contrived sample) that have been subjected to a liquid biopsy assay, quantified, and then contributed by members of the BloodPAC Consortium. In all, there are more than 31,000 files in the commons as of December 2020. We describe the BPDC, the data it manages, the process that the BloodPAC Consortium used to develop it, and some of the applications that have been developed using its API.

**CONCLUSION:**

The BPDC has been the data platform used by BloodPAC during the past 4 years to manage the data for the consortium and to provide workspaces for its working groups.

## INTRODUCTION

The science and applications concerning liquid biopsies are emerging and rapidly evolving. In this paper, a liquid biopsy is defined as the analysis of cell-free DNA (cfDNA), circulating tumor DNA (ctDNA), extracellular vesicles, and/or circulating tumor cells (CTCs) that are obtained by minimally invasive routine blood draws. For many years, the concept of the liquid biopsy has been considered the holy grail of medical oncology. This is due to the many immediate clinical applications and the speed and safety compared with traditional tissue-based biopsy methods (see, for example, Table 1 in Aggarwal et al^1^). One of the challenges with liquid biopsy technologies is the need to capture, manage, and harmonize large amounts of digital genomic, molecular, and cellular data and the associated clinical data. Thus, there is a need for a data commons to address these challenges and to become a hub of a learning and evolving cancer data ecosystem that supports basic, regulatory, and clinical research endeavors.

**CONTEXT**

**Key Objective**
To develop a data commons to support the activities and aims of the Blood Profiling Atlas in Cancer (BloodPAC) Consortium, a public-private partnership whose goal is to accelerate the development, validation, and clinical use of liquid biopsy assays to improve patient outcomes for patients with cancer.
**Knowledge Generated**
By harmonizing disparate data contributed by BloodPAC stakeholders to a consensus data model and framework, the BloodPAC Consortium has leveraged the BloodPAC Data Commons and published (1) recommended preanalytic technical data elements and (2) a generic protocol for designing analytical validation studies of next-generation sequencing–based circulating tumor DNA assays.
**Relevance**
The BloodPAC Data Commons has laid the initial foundation to serve as one of the sources of valid scientific evidence to advance the field of liquid biopsies and its applications to improving cancer outcomes.

A data commons co-locates (1) data, (2) cloud-based storage and computing infrastructure, and (3) commonly used software services, applications, and workspaces to create a resource for a community.^2^ In 2017, a public-private partnership called the Blood Profiling Atlas in Cancer (BloodPAC) Consortium launched a data commons called the BloodPAC Data Commons (BPDC) to support the greater scientific research, regulatory, and clinical communities in developing liquid biopsy assays with the goal and mandate to improve outcomes for patients with cancer.^3^ In this article, we describe the BPDC, the process that the BloodPAC Consortium used to develop BPDC, and some of the applications that have been developed over BPDC.

The BloodPAC Consortium was started to address a number of issues related to liquid biopsies, including furthering the generation of evidence to bring liquid biopsy into routine clinical practice. BloodPAC is organized into working groups that address questions such as what are the minimum data elements required when liquid biopsy data are collected, what are analytical validation protocols for developers or manufacturers of next-generation sequencing–based ctDNA diagnostic tests, and what is the concordance between liquid and solid biopsies? The first two questions were addressed by BloodPAC Working Groups and resulted in consensus recommendations, summarized by a BloodPAC technical publication.^4,5^ The third question is being addressed by BloodPAC's Project Exhale, which is currently collecting relevant data from BloodPAC members.

**FIG 1.**
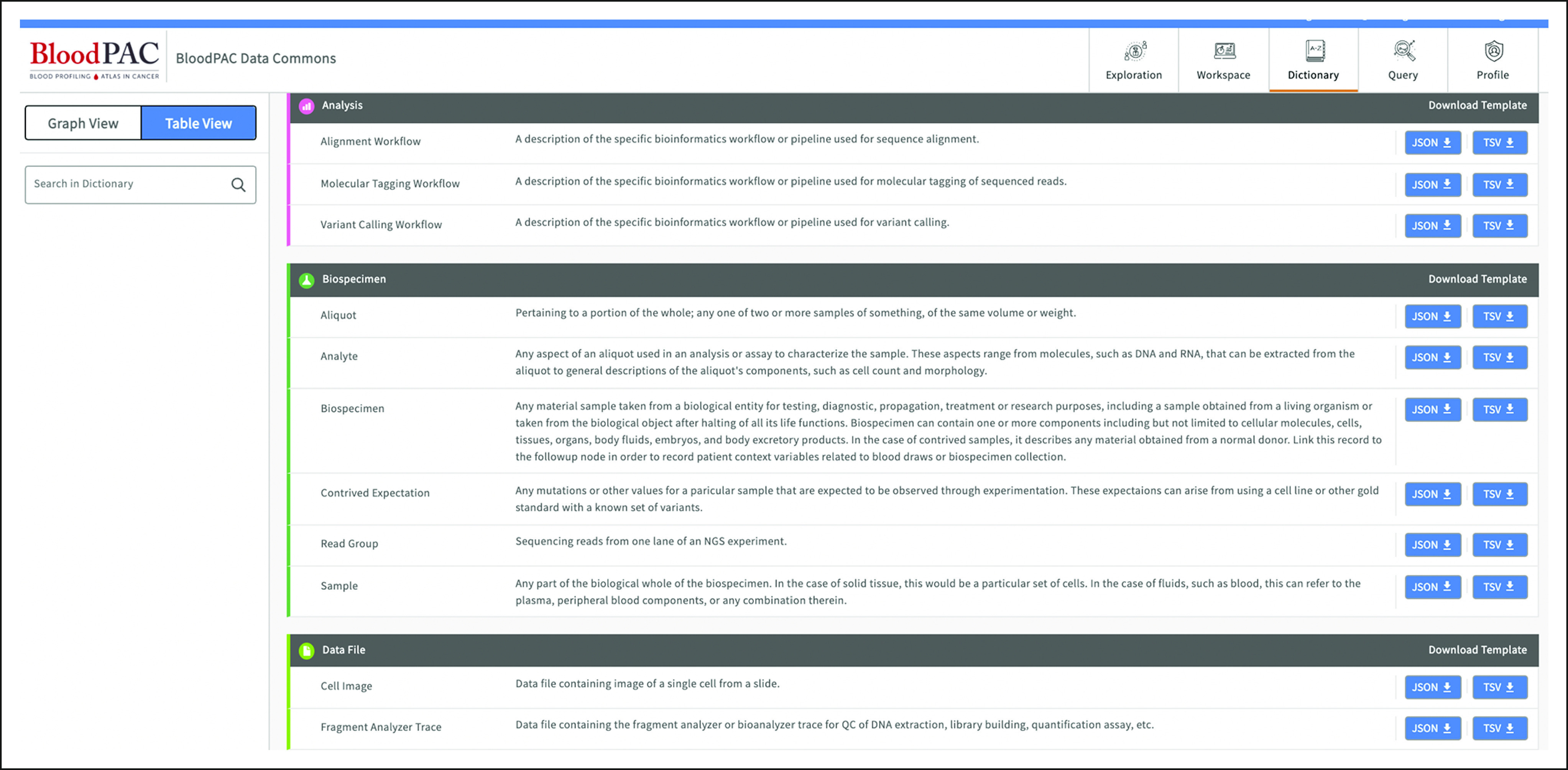
The BloodPAC Data Commons data model. BloodPAC, Blood Profiling Atlas in Cancer; UAMS, University of Arkansas Medical Sciences; USC, University of Southern California.

The BPDC^6^ consists of several connected components: (1) the BPDC Governance Structure that includes agreements for contributing, accessing, and analyzing data in the BPDC; (2) the BPDC platform itself that includes an API that supports BloodPAC and third-party applications; (3) the BloodPAC Data Model (Fig 1) that describes data managed by the platform; (4) data that are contributed and managed by the commons; and (5) applications that are built over the commons that accesses data from the commons using the BPDC API and associated BPDC services. This paper describes components (2), (3), (4), and (5). BloodPAC uses the Open Commons Consortium (OCC) Data and Commons Governance Framework for (1).^7^

## RESULTS

### BloodPAC Data

The BPDC contains 57 studies and projects spanning more than 4,100 cases. This amounts to 5,700 aliquots (blood plasma, serum, or a contrived sample) that have been subjected to a liquid biopsy assay, quantified, and then contributed by members of the BloodPAC Consortium. In all, there are more than 31,000 files in the commons as of December 2020.

The BPDC hosts data sets from a variety of assay types that are used for different purposes. Each type of assay is represented in the commons as a separate table, including immunoassays, sequencing assays, polymerase chain reaction assays, quantification assays, and mass cytometry assays. Each of these tables include experimental metadata to facilitate finding and subsetting data across individual data sets. Users can use these metadata elements to perform faceted search of data sets, patients, and files and download the tables for their selected cohorts.

Data contributors are required to submit experimental metadata on the instruments and platforms used for assays. Data contributors are encouraged but not required to submit accompanying metadata for library preparation kit, target capture kit, and assay kit names, vendors, and versions. These data are largely provided where relevant.

Data contributors are encouraged to provide both raw and processed data so that future analyses can reprocess the raw data using novel techniques or pipelines. For example, among the contributed 2,625 submitted somatic mutation variant call format files, 64% have corresponding unaligned reads files (FASTQ), aligned reads files, or both.

### BloodPAC Data Model

**FIG 2.**
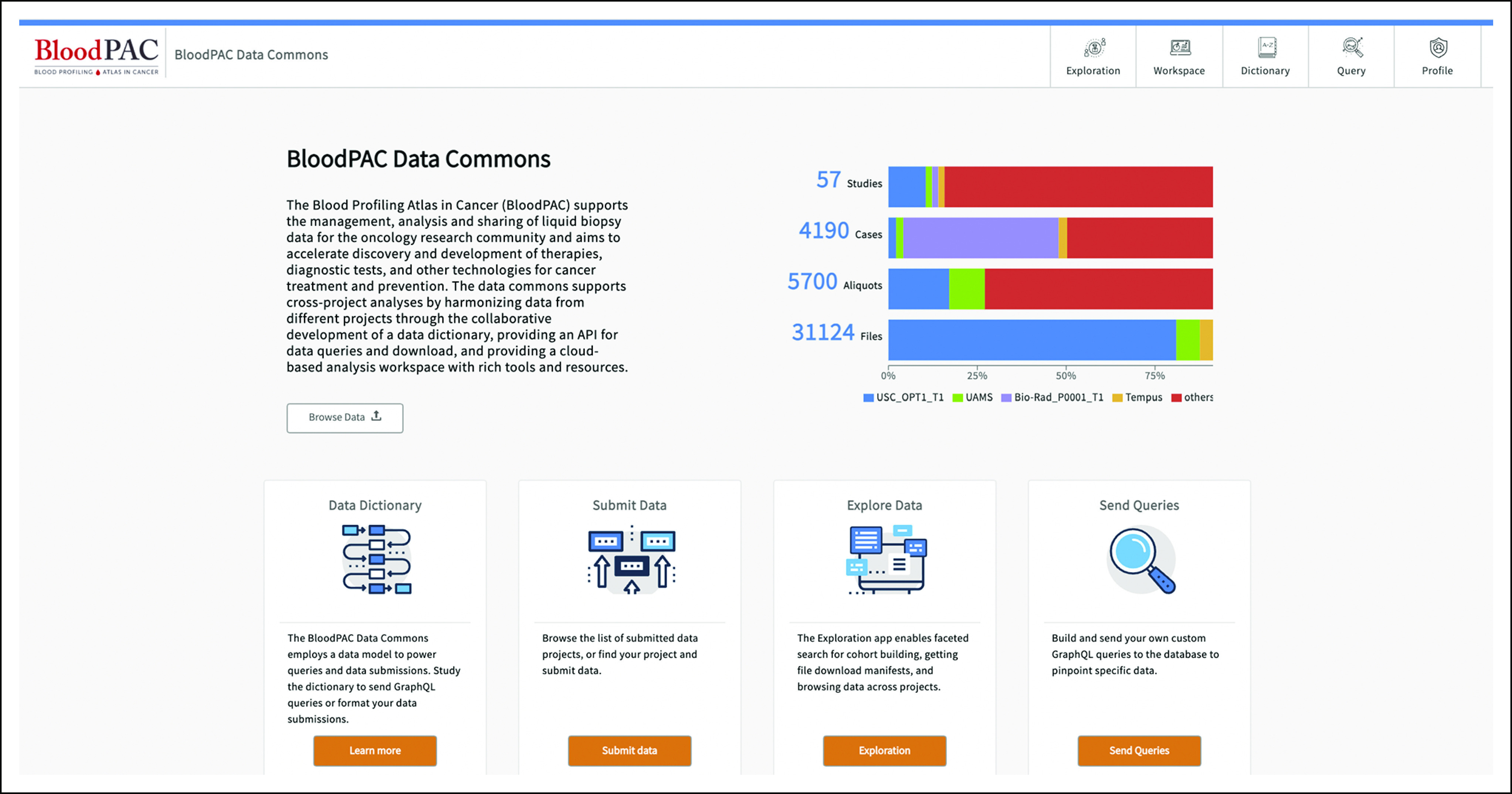
The BloodPAC Data Commons portal. BloodPAC, Blood Profiling Atlas in Cancer.

The data for all the studies are curated using a common data model (BloodPAC Data Model). The current version is 0.7.5, which is the 38th release of the model. Liquid biopsy data beyond genomic sequencing (eg, CTCs and extracellular vesicles) have been added to the model since its inception. The current data model is a graph-based data model with 44 nodes, 72 edges, and more than 380 attributes. The data model can be visualized and explored using the BPDC portal as shown in Figure 2.

Working iteratively for over a year, a BloodPAC working group developed minimum technical data elements (MTDEs) that are required for any future cfDNA data that are accepted by the BPDC^4^ after January 1, 2018.

### BPDC Platform

The BPDC is based upon the Gen3 Data Commons platform^8^ that was developed by the Center for Translational Data Science at the University of Chicago and includes (1) a Data Exploration Portal that supports interactive exploration of the data and the creation of synthetic cohorts; (2) a Data Submission Portal and data submission API; (3) virtual machine–based workspaces, container-based workspaces, and Jupyter notebook–based workspaces for exploring the data, including virtual cohorts created using (1); (4) a Data Dictionary that supports graphical and tabular views; and (5) a portal for managing user security credentials to access the workspaces (3). A view of the BloodPAC Data Exploration portal is shown in Figure 2.

### BPDC Applications

In this section, we describe three applications that have been built over the BPDC to illustrate the types of applications that are possible using the BPDC API. As background, a primary aim of the BPDC is to provide the cancer research community with a data platform that facilitates data sharing and accelerates liquid biopsy assay research and development. As mentioned above, the BloodPAC Consortium developed preanalytical MTDEs that are required for all cfDNA data that are submitted to the BPDC.^4^ The three applications described below all use the MTDEs.

#### Application 1

**FIG 3.**
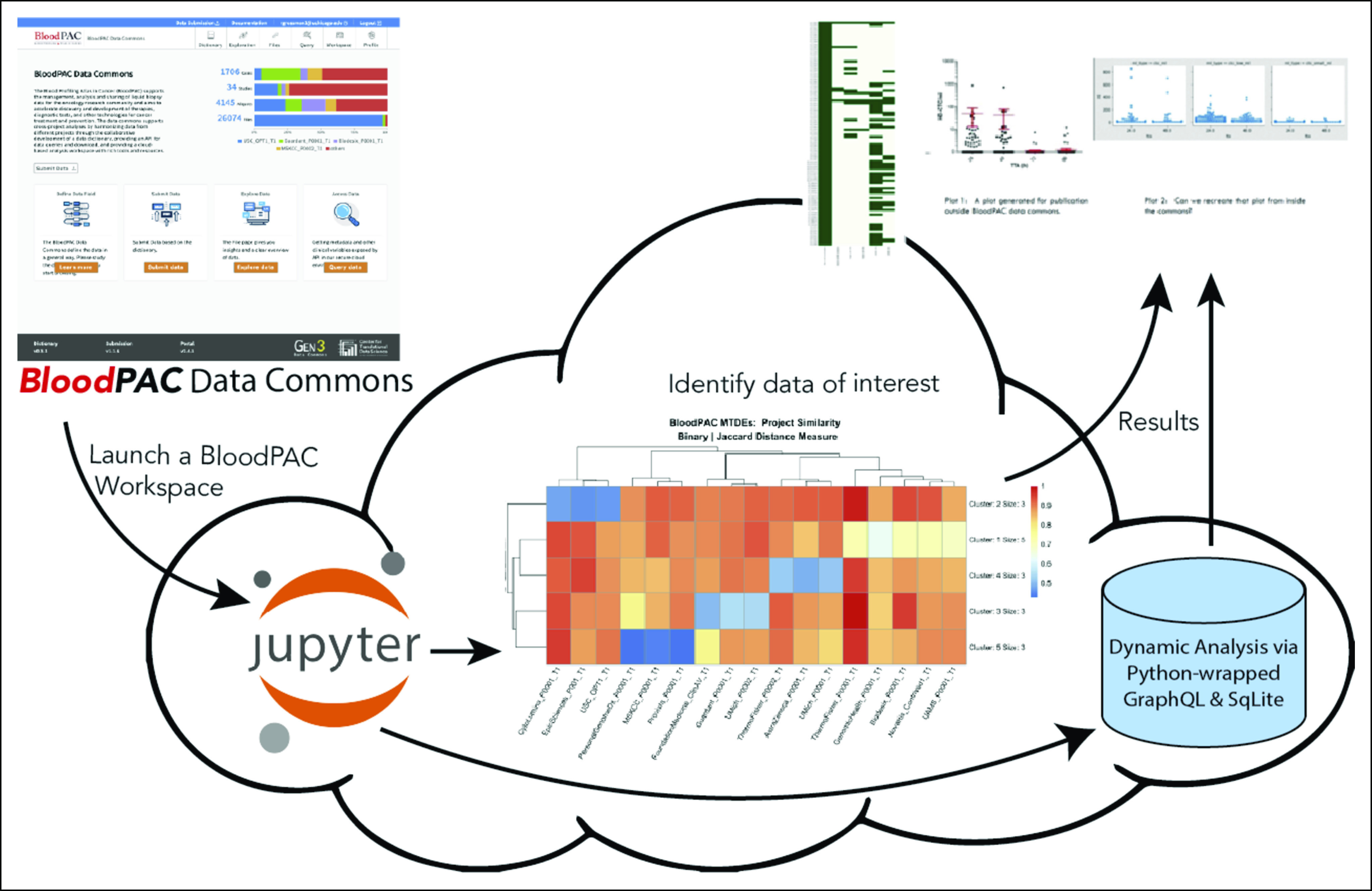
The figure shows how applications can be built over the BloodPAC Data Commons API. The heatmap and plots described for application 3 can be seen at the top right of this image highlighted by the dashed rectangle. BloodPAC, Blood Profiling Atlas in Cancer; MTDE, minimum technical data elements; UAMS, University of Arkansas Medical Sciences; USC, University of Southern California.

The first application accessed the preanalytical MTDE data from the commons and clustered them to visualize the similarity, or lack of similarity, of the MTDE data across the data from the various projects submitted to the BPDC. The counts data for each preanalytical MTDE were pulled for each project and aggregated to create a matrix of counts. Since the counts matrix was sparse and since there was a high variance in count magnitude between projects, the counts were transformed into binary attributes, with a 1 for those elements that were ≥ 1 and 0 otherwise. Using the binary count matrix, the Jaccard measure was computed to create a distance matrix. That distance matrix was then run through a k-means clustering algorithm to create a heatmap, indicating which projects had the most similar preanalytical MTDE submissions. Figure 3 in the middle shows the heatmap. The source code for this example can be found in Ref. 9. This application was developed by the OCC, one of the BloodPAC members.

#### Application 2

The second application illustrates how the BPDC can be used to perform custom ad hoc queries over the data it hosts. This application was also used as a quality check of the standard operating procedure (SOP) to submit and process data since the results of the analysis were compared with the results of the analysis done locally by the BloodPAC member that contributed the data. After an authenticated login into the BPDC, a virtual machine was launched and a Jupyter notebook was created. Using the preanalytical MTDEs^4^ and a GraphQL query executed through Python, a test set of eight patients were identified and DNA panel data and RNA-seq data associated with the patients were accessed and copied to the virtual machine. Pysam, a Python wrapper for SAMtools,^10^ was then used to access specific gene mutations from the variant call format (VCF) files. The results matched a similar analysis done locally on the original data set. A SQLite database was then created within the BPDC and loaded with the VCF data from the BPDC. The database was about 1 MB in size. Queries to the database to find patients with specific mutations of interest (eg, *MUC16*) were compared with those done locally on an internal database. Again, the results were identical, and response time was comparable. This application was developed by the University of Arkansas Medical Sciences, one of the BloodPAC members. Source code for this example can be found in Ref. 11.

#### Application 3

Application 3 was developed by the University of Southern California, another BloodPAC Member. In a previously published manuscript,^12^ they analyzed the effect of preanalytical variables (eg, time to analysis) on the enumeration of rare cells in blood. The data for this study were contributed to the BPDC. This application queried the BPDC to produce a plot showing the available multianalyte experimental results from a set of patient samples. The rare cell enumeration, genomics, and proteomics data available per patient sample in the BPDC are shown as a green heatmap at the top right of Figure 3. In addition, a Jupyter Notebook in a BPDC workspace was used to recreate a rare cell enumeration plot of patient samples processed at the 24 versus 48 hour time-to-analysis time points using the preanalytical MTDE of Maximum Time to Fractionation. The plot produced can be seen at the top right of Figure 3 to the right of the heatmap. The plot of the CTC enumeration for the matched 24- and 48-hour patient blood samples generated by a query against the BPDC matched closely the same plot previously produced for the publication.^12^ This application demonstrated that it is possible to regenerate plots using the data commons that is similar to the ones produced previously by the research team in their own lab. This is important not only to demonstrate that effective querying is available for the data in the commons, but also that others in the scientific community can perform the same analysis described in manuscripts using the BPDC. The Jupyter notebooks containing the python source code for these examples can be found in Ref. 13.

## METHODS

### Setting Up the BPDC

The BPDC is implemented using the open source Gen3 data commons platform.^8^ In particular, the BPDC Data Exploration Portal, BPDC Data Submission Portal, the BPDC Workspace Hub, and the BloodPAC API were all automatically generated from the BloodPAC Data Model using the Gen3 data commons platform. BPDC uses Gen3's implementation of the data commons framework services^2^ so that it can interoperate through secure, compliant APIs with other data commons using data commons framework service, such as National Cancer Institute's Cancer Research Data Commons.^14^

The process used to set up the BPDC consisted of the following steps:

1. BloodPAC agreed to a set of data and commons governance documents for submitting data to the commons, accessing data from the commons, and analyzing data in the commons. BloodPAC used the Data Commons Governance Framework developed by the not-for-profit OCC.
2. BloodPAC developed a set of research questions that were precompetitive and important to its members to provide a focus for the development of the BPDC.
3. BloodPAC set up a working group to develop a data model for data uploaded and managed by the Commons.
4. BloodPAC set up a working group to establish MTDEs for data submitted to the BPDC.
5. BloodPAC organized a set of what it called *data trains*, so that data could be uploaded to the BPDC, whereas (2), (3), and (4) were iteratively refined.
6. Data sets contributed to BloodPAC were organized around specific scientific questions. Several BloodPAC members developed Jupyter notebooks and simple applications over the BPDC API that analyzed the data in the BPDC and produced figures that were suitable for publication showing the results of their analysis. This demonstrated the value of the BPDC to the broader BloodPAC membership and the importance of contributing data to it.

### Development of the BloodPAC Data Model

As mentioned, data were imported into the BPDC iteratively in stages called data trains. In Data Train 1, BloodPAC came to an initial consensus about the minimum preanalytical data fields required and the importance of carefully defining these fields, making sure that all data submissions included all of them. These came to be known as the preanalytical MTDEs. Later, data trains were required to use the MTDEs, and an effort was made to go back and obtain the MTDEs for those data sets that did not have them.

The BPDC data dictionary was originally developed using the core clinical and biospecimen nodes from the Genomic Data Commons (GDC) dictionary.^15^ Although most of the first nodes and properties were identical to the GDC dictionary, the data model was extended to include blood biopsy–specific nodes and properties. Small changes were made in the first few months to allow users to submit their data as part of Data Train 1. The original BPDC dictionary was used for about 6 months before receiving a substantial reorganization of nodes and properties based upon the experience from uploading data in Data Train 1. A major release of the data dictionary was developed and used for Data Train 2. The key changes were related to the simplification of the biospecimen tree and expansion of data file nodes. Since the move to the second version, the changes to the data dictionary have mirrored those from the first few months—small changes and additions to allow users to submit their data. Some of the changes include more detailed blood biopsy–specific clinical data achieved through changes to the links and properties on the clinical nodes, new workflow nodes to detail analytical validation, and other processing done on data files. The data dictionary is open access and is available in Ref. 16.

### Governance Structure

The BPDC governance structure is based upon the OCC governance structures, policies, and agreements. BloodPAC and the OCC are part of the same 501(c)(3) not for profit. The OCC principles, policies, and agreements are all available in Ref. 17, including consortium membership agreements, data contributors agreements, data access agreements, security and compliance agreements, intellectual property agreements, and publication policies. BloodPAC's versions of these agreements are very similar and can be downloaded from the BloodPAC website or requested by contacting info@bloodpac.org.

As mentioned above, the BPDC is based upon the Gen3 data platform. It is operated by the Commons Services Operations Center at the Center for Translational Data Science at the University of Chicago. The Gen3 data platform and associated Commons Services Operations Center SOPs follow the policies, procedures, and controls for a Moderate system as described in NIST SP 800-53. In addition, there are periodic independent assessments by a third party and a yearly penetration test by a third party. All data submitted to the BPDC are deidentified and so are not human subject data and regulated by Health Insurance Portability and Accountability Act.

Data are submitted to the BPDC using the BloodPAC Data Contributors agreements, which require patient consent be obtained or an appropriate process (such as a one-time waiver of consent by the institutional review board) be used instead. It is the responsibility of the data contributor to obtain the required patient consents. If the BPDC is informed by the Data Contributor of withdrawn data, then the BPDC will remove the data, but not always its inclusion in already published data or aggregated data.

### Sustainability

The current sustainability model for the BloodPAC Commons is for members through member contributions to support the core operations, including data storage, of the BPDC, and for users accessing the data via workspaces to pay for their workspaces and the associated computing costs. Finally, liquid biopsy data hosted for the public are chosen to balance the significance and importance of the data with their size, since by accepting the data, the BPDC assumes a long-term responsibility for hosting the data.

## DISCUSSION

Liquid biopsies bring a promise of improved care for patients with cancer and are now beginning to produce a disruptive change regarding clinical oncology, patient management, and the design of cancer therapeutics. Per the 21st Century Cures Act, innovative clinical trials are integral for biomarker and drug development.^18^ The FDA Oncology Center of Excellence has highlighted the importance and pursuit of novel clinical trial designs incorporating blood-based biomarkers such as ctDNA in their recent annual report.^19^ But the continuous validation, deployment, translation, and contextualization of the challenging digital data streams germane to the liquid biopsy may all be met and streamlined by the fruits of a robust BPDC.

The BPDC can be viewed as a publicly accessible database that contains valid scientific evidence about certain questions of interest to the liquid biopsy community (compare ref. 20). BloodPAC is developing SOPs and protocols that describe how data in the BPDC are collected, processed, curated, and evaluated. For BloodPAC studies, the BPDC captures sufficient metadata and has in place data quality and other controls so that the data products produced for the studies are reproducible and sufficiently documented so that they could be in principle reproduced by third parties.

BloodPAC is operated using policies, procedures, and controls that are designed to protect the confidentiality, integrity, and availability of the submitted data. Although data in the BPDC are deidentified, the security, privacy policies and controls that BloodPAC uses are designed to protect sensitive data, such as data containing protected health information.

## APPENDIX BloodPAC Executive Committee and Scientific Co-Chairs

### Executive Committee

Philip G. Febbo—Senior Vice President and Chief Medical Officer, Illumina

Robert L. Grossman—Professor, University of Chicago CTDS and Founder/Director, Open Commons Consortium

Peter Kuhn—Professor, University of Southern California

Anne-Marie Martin—Senior Vice President, Global Head, Experimental Medicine Unit, GSK

Jake Vinson—Chief Executive Officer, Prostate Cancer Clinical Trials Consortium

### Scientific Co-Chairs

Kelli Bramlett—Director of R&D, Thermo Fisher Scientific

Darya Chudova—Senior Vice President, Technology, Guardant Health

James H. Godsey—VP, Assay Development, Illumina

Jerry S. H. Lee—Chief Science and Innovation Officer, Lawrence J. Ellison Institute for Transformative Medicine of University of Southern California

Hakan Sakul—VP and Head of Diagnostics, Pfizer

Howard I. Scher—Physician and Head, Biomarker Development Initiative at Memorial Sloan Kettering Cancer Center

Jennifer Dickey, Vice President, Regulatory and Quality, Personal Genome Diagnostics

### BloodPAC Consortium Collaborators

1. American Association for Cancer Research (AACR)
2. American Cancer Society (ACS)
3. Association for Molecular Pathology
4. AstraZeneca
5. Bio-Rad Laboratories
6. Breast Cancer Research Foundation (BCRF)
7. Bristol-Myers Squibb
8. Center for Translational Data Science at the University of Chicago
9. Center for Genetic Medicine Research at Children's National Medical Center
10. Ceres Nanosciences
11. Chan Soon-Shiong Institute of Molecular Medicine at Windber
12. College of American Pathologists
13. Eli Lilly and Company
14. Epic Sciences
15. Fluxion Biosciences
16. Focused Ultrasound Foundation
17. Foundation for the National Institutes of Health
18. Foundation Medicine, Inc
19. Freenome
20. Friends of Cancer Research
21. GlaxoSmithKline
22. Guardant Health
23. Horizon Discovery Ltd
24. Illumina, Inc
25. Inivata
26. LGC/Seracare
27. Memorial Sloan Kettering Cancer Center (MSKCC)
28. Movember Foundation
29. National Cancer Institute at the National Institutes of Health (NIH/NCI)
30. Novartis
31. Open Commons Consortium (OCC)
32. Personal Genome Diagnostics (PGDx)
33. Pfizer, Inc
34. Prostate Cancer Foundation (PCF)
35. SolveBio
36. Streck
37. Sysmex Inostics
38. Tempus Labs
39. The Arkansas Bioinformatics Consortium (AR-BIC)/University of Arkansas
40. The Prostate Cancer Clinical Trials Consortium (PCCTC)
41. Thermo Fisher Scientific
42. University of Southern California
43. US Department of Defense (DOD)
44. US Department of Veteran's Affairs (VA)
45. US Food and Drug Administration (FDA)
